# Assessment of the Relationship Between Executive Function and Cardiorespiratory Fitness in Healthy Older Adults

**DOI:** 10.3389/fpsyg.2021.742184

**Published:** 2021-11-03

**Authors:** David Predovan, Nicolas Berryman, Maxime Lussier, Francis Comte, Thien Tuong Minh Vu, Juan Manuel Villalpando, Louis Bherer

**Affiliations:** ^1^Centre de Recherche, Institut Universitaire de Gériatrie de Montréal, Montréal, QC, Canada; ^2^Département de Psychologie, Université du Québec à Montréal, Montréal, QC, Canada; ^3^Centre de Recherche, Institut de Cardiologie de Montréal, Montréal, QC, Canada; ^4^Département des Sciences de l’Activité Physique, Université du Québec à Montréal, Montréal, QC, Canada; ^5^Département de Médecine, Université de Montréal, Montréal, QC, Canada; ^6^Département de Médecine, Centre Hospitalier de l’Université de Montréal, Service de Gériatrie, Montréal, QC, Canada; ^7^PERFORM Centre, Concordia University, Montréal, QC, Canada

**Keywords:** aging, cardiorespiratory fitness, cognition, executive function, physical activity

## Abstract

Associations between cardiorespiratory fitness and brain health in healthy older adults have been reported using a variety of cardiorespiratory fitness estimates (CRFe). Using commonly used methods to determine CRF, we assessed the relationship between CRFe and executive function performance. Healthy older adults (*n* = 60, mean age 68 years, 77% women), underwent three CRF tests: a Maximal Graded Exercise Test performed on a cycle ergometer, the Rockport Fitness Walking Test, and a Non-Exercise Prediction Equation. Executive function was assessed by a computerized cognitive assessment using an N-Back task (updating cost) and a Stroop task (interference cost, global and local switch cost). Multiple hierarchical regression analyses were conducted to assess the relationship between different CRFe and executive function performance. Regardless of age and education, cardiorespiratory fitness estimated from the Maximal Graded Exercise Test and the Rockport Fitness Walking Test was significantly associated with the global switch cost. All CRFe were associated with the interference cost. No association was observed between CRFe and local switching costs or the updating costs. In the present study, not all subcomponents of executive function were related to CRFe. Interestingly, the executive functions that were associated with CRFe are those that are known to be the most affected by aging.

## Introduction

The long-term benefits of lifelong physical activity (Larson et al., 2006), particularly on cognitive aging (Gajewski and Falkenstein, 2015a; Berryman et al., 2018), have been extensively studied. However, more studies are needed to determine how physical activity interacts with brain health. One potential mechanism is cardiorespiratory fitness (CRF; Hayes et al., 2013). CRF, expressed as $\dot{V}\,O_{2}\,m\,a\,x$, represents the body’s maximal ability to transport and use oxygen, from the atmosphere to the working muscles (Ross et al., 2016). Integrating multiple physiological systems (pulmonary ventilation, cardiovascular function, and muscular work), CRF is positively associated with multiple health outcomes, such as cognitive functions (Hayes et al., 2016), as well as future cognitive states (Wendell et al., 2014). In older adults, participation in both acute and long-term moderate- to vigorous-intensity physical activity, such as aerobic exercise (known to increase CRF), has been shown to improved brain function (Erickson et al., 2019).

In the context of aging research, a Maximal Graded Exercise Test (GXT), the standard procedure for evaluating $\dot{V}\,O_{2}\,m\,a\,x,$ may not be feasible as it requires critical screening procedures and medical supervision to optimize safety (Huggett et al., 2005). To overcome these practical issues, the use of submaximal exercise tests [such as the Rockport Fitness Walking Test (ROCKPORT) or a Non-Exercise Prediction Equations (EQUATION)] may be recommended, although they are likely to be less precise than a GXT. For example, CRF estimate (CRFe) obtained with EQUATION, is often based on self-reported measures that may be subject to memory problems, social desirability, and participant subjectivity, which could lead to measurement errors (Wang et al., 2019).

In older adults (*n* = 86, M age 65.14, 61.6% women), a cross-sectional study found significant associations between three CRFe (namely a GXT performed on a treadmill, the ROCKPORT, and EQUATION) and working memory, processing speed, and memory complaints (McAuley et al., 2011). In their report, the authors emphasized the importance of further research to better quantify potential associations between executive function (EF) and CRFe, particularly EQUATION. EF is required when the behavior or information processing to be performed is not automated and required some control (Klein, 1996). This control may be necessary because of the unpredictability or novelty of the situation (Banich, 2009). EF is also needed when two tasks cannot be processed in parallel in working memory (de Fockert et al., 2001) or consecutively without affecting performance (flexibility or *switching*). The second characteristic of EF is that they are initiated proactively, voluntarily (even consciously) (Lezak, 1995), and toward a goal (Miller and Cohen, 2001) such as solving a problem, carrying out a predetermined plan (Unterrainer et al., 2004) or optimizing performance on a task. Remembering a phone number for a few minutes would require such control given the continual *updating* of information in working memory (Shiffrin and Schneider, 1977). This control might go against an automated process (Shallice, 1988) (e.g., *inhibition* of an automated response such as reading a word in a Stroop task, where one must instead name the color of the ink with which the word is written).

Several studies conceive EF as a “fractionable” concept (Alvarez and Emory, 2006), which relies on distinct cognitive mechanisms of attentional control. EF can be conceptualized as a three-factor model (Friedman and Miyake, 2017) that includes *switching* (local and global), *inhibition*, and working-memory *updating*. Some studies suggest that the subcomponents of EF are affected differently by aging. For instance, updating tends to be less affected by age compared with inhibition and switching (Maldonado et al., 2020). Moreover, in normal aging, the decline in global switching (e.g., the cost of maintaining and coordinating two tasks relative to performing a single task) is known to be more pronounced and to occur earlier compared to local switching (e.g., the cost associated with alternating between two tasks) (Kray and Lindenberger, 2000; Wasylyshyn et al., 2011; Kray and Dörrenbächer, 2019).

As $\dot{V}\,O_{2}$ max has been shown to decrease with age (Hawkins and Wiswell, 2003), many studies have investigated its relation to EF in older adults. Understanding the relationship between CRF and EFs is of particular interest, as they are known to predict older adults’ ability to maintain independent living (Vaughan and Giovanello, 2010) and has been associated with many behaviors that affect daily life, including driving (Anstey et al., 2005), walking (Mirelman et al., 2012), medication adherence (Insel et al., 2006), urinary incontinence (Lussier et al., 2013), and use of problem-solving strategies (Taconnat and Lemaire, 2014). EF was associated with the Instrumental Activities of Daily Living (IADL) scale (Cahn-Weiner et al., 2002) and was associated with a measure of life expectancy and quality of life (Quality Adjusted Life Year) (Davis et al., 2010) in older women.

Association has been reported in older adults between EF and GXT CRFe (van Boxtel et al., 1997; Barnes et al., 2003; Brown et al., 2010; Verstynen et al., 2012; Weinstein et al., 2012; Berryman et al., 2013; Albinet et al., 2014; Freudenberger et al., 2016). Moreover, an association between a z-composite score combining the CRFe provided by the ROCKPORT and a GXT performed on a treadmill was found with EF (Prakash et al., 2011). Performance on the ROCKPORT was also directly associated with inhibition as measured by a flanker task (Colcombe et al., 2004). In a study assessing physical activity levels through questionnaires and accelerometers, CRF with the ROCKPORT and EF with a three-factor model (Miyake et al., 2000) (but only using a global switching task), only inhibition was associated with physical activity (Boucard et al., 2012). As CRF was not measured by GXT, the lack of association between performance on the global switching task and CRFe could have been explained by the choice of CRFe. To our knowledge, no study has used all types of CRF estimation to assess potential associations with EF, and few studies have examined all subcomponents of EF. To address this shortcoming, the objective of this study was to assess in older adults, CRFe (GXT, ROCKPORT, EQUATION) and their association with EF [switching (both local and global), inhibition, and working-memory updating]. Furthermore, to minimize the task impurity that characterized EF task (Friedman and Miyake, 2017) and to reduce the effect of processing speed on EF (Albinet et al., 2012), performance for each EF was computed in terms of different costs calculated in percentages. We hypothesized that the EF subcomponents (inhibition and global switching) known to decline the most during aging would be strongly associated with the most precise CRFe (GXT and, to a lesser degree, ROCKPORT). No significant association is expected between EF subcomponents that are known to be less altered during aging such as updating and local switching.

## Materials and Methods

### Experimental Procedures

As a screening procedure to optimize participants’ safety, each participant was assessed by a geriatrician before CRF testing. The CRF tests [GXT, ROCKPORT, and EQUATION (Jurca et al., 2005)] were conducted on two non-consecutive days (7.15 ± 1.66 days apart). On the first day of the assessment, participants completed a neuropsychological and a geriatric assessment, followed by a GXT. On the second day, participants completed a questionnaire assessing their habitual physical activity levels (Self-Reported Physical Activity) to complete the EQUATION, and then proceed with the ROCKPORT.

### Participants

This study was approved by the Ethics Committee of the geriatric institution where it was conducted and was part of a larger clinical trial (NCT02455258). Informed consent was obtained from all participants. Participants were recruited from the community through advertisements in newspapers, magazines dedicated to the older adults, flyers, websites, and through the research center’s pool of participants. To be included, participants had to be sedentary (less than 150 min of moderate-intensity exercise per week) and at least 60 years old. Exclusion criteria were the use of a walking aid or hormone therapy, having undergone surgery under general anesthesia, participation in a structured physical activity program within the past 6 months, smoking within the past 5 years, having been diagnosed and/or treated for major depression or uncontrolled medical conditions such as neurological disorders and cardiorespiratory diseases, and presenting significant or uncorrected perceptual limitations or significant cognitive impairment [as determined by a cutoff score of 26 on the Mini-Mental State Examination (Folstein et al., 1975)]. Table 1 presents the characteristics of the participants (*n* = 60, mean age 68 years, 77% women).

**TABLE 1:** Participants’ characteristics, cardiorespiratory fitness, and cognitive data.

Characteristic	
Age	67.65 ± 5.42
Sex	14 (M)/46 (W)
Education (years)	15.20 ± 3.54
Mini-Mental State Examination	27.98 ± 1.55
Body Mass Index	26.82 ± 4.80

**Cardiorespiratory fitness**	

Maximal graded exercise test (ml.kg^–1^.min^–1^)	21.24 ± 4.51
Rockport Fitness Walking Test (ml.kg^–1^.min^–1^)	18.31 ± 7.57
Non-exercise prediction equation (ml.kg^–1^.min^–1^)	24.38 ± 6.52
Rockport Fitness Walking Test completion time (s)	1095.02 ± 105.96
Heart Rate (bpm; rest)	66.47 ± 11.71
Heart Rate (bpm; after Rockport Fitness Walking Test)	117.00 ± 17.17
Self-Reported Physical Activity Questionnaire (score 0 to 4)	1.61 ± 1.18

**Cognition: Updating task**	

N-Back 1 RT (ms)	1014.08 ± 176.22
N-Back 2 RT (ms)	1242.34 ± 254.06
N-Back 1 accuracy (%)	89.5
N-Back 2 accuracy (%)	62.5
Updating cost (%)	23

**Cognition: Stroop task**	

Reading condition RT (ms)	1035 ± 155.56
Counting condition RT (ms)	1054.45 ± 165.84
Inhibition RT (ms)	1194.97 ± 188.82
Switching RT (ms)	1545.88 ± 277.08
Reading condition accuracy (%)	96.32
Counting condition accuracy (%)	96.26
Inhibition accuracy (%)	96.93
Switching accuracy (%)	92.67
Interference cost (%)	14
Global switching cost (%)	21
Local switching cost (%)	19

### Assessment of Cardiorespiratory Fitness

Under the supervision of a geriatrician and a certified kinesiologist, GXT was performed on a cycle ergometer (Corival Recumbent, Lode B.V., Groningen, Netherlands) with a metabolic cart (Moxus, AEI Technologies Inc., Naperville, IL, United States). The full protocol has been described elsewhere (Berryman et al., 2013, 2014). The test started at 35W for women and 50W for men. Participants were instructed to maintain a pace between 60 and 80 rpm. Each minute, the power of the ergocycle was increased by 15W. The test ended when the participant was unable to maintain the required pace. Constant positive verbal encouragement was given during the test to ensure maximal effort from the participant. The highest $\dot{V}\,O_{2}$ over a 30-s period was considered as $\dot{V}\,O_{2}$ peak (in ml.kg^–1^.min^–1^). All analyses for the GXT included only participants who achieved a respiratory exchange ratio (RER) greater than 1.1, a value that qualifies participants’ effort during the test as excellent (Balady et al., 2010).

For the ROCKPORT, participants were asked to walk one mile (1,609 m) as fast as possible (Fenstermaker et al., 1992) on an indoor track. Participants performed this test alone or in small groups of two to four people. A stopwatch was used to record the time achieved and heart rate was recorded throughout the test by a Polar RS 800 beat-to-beat recorder (Polar Electro Oy, Kempele, Finland). CRF was estimated using an equation that included age, biological sex, body mass, time to complete the ROCKPORT, and post-ROCKPORT heart rate (Kline et al., 1987; Fenstermaker et al., 1992). ROCKPORT CRFe in ml.kg^–1^.min^–1^ = 132.853 – {0.0769 × [Weight (lb)]} – {0.3877 × [Age (Year)]} + {6.315 × [Sex (Men = 1, Women = 0)]} – {3.2649 × [Walked Time (min)]} – {0.1565 × [Post Exercise Heart Rate (beats per minute)]} (Kline et al., 1987).

Cardiorespiratory fitness was also estimated with the equation derived by Jurca et al. (2005): EQUATION. This equation was designed to consider sex, age, Body Mass Index, and resting heart rate, as well as self-reported habitual physical activity levels [as assessed by a Self-Reported Physical Activity questionnaire (SRPA)]. EQUATION CRFe in ml.kg^–1^.min^–1^ = [Sex (Men = 1, Women = 0)] × (2.77) – Age × (0.10) – Body Mass Index × (0.17) – Resting Heart Rate × (0.03) + SRPA + 18.07. The SRPA categorizes the participant’s self-reported level of physical activity. Category 1: Inactive or low levels of physical activity other than usual daily activities (value = 0). Category 2: Regular (≥5 days/week) participation in physical activities for at least 10 min at a time and requiring low level of exertion (value = 1). Category 3: Engagement in aerobic exercises at a comfortable pace for 20–60 min per week (value = 2). Category 4: Participation in aerobic exercises at a comfortable pace for 1–3 h per week (value = 3). Category 5: Participation in aerobic exercises at a comfortable pace for over 3 h per week (value = 4). Table 1 presents participants’ results on all CRF assessments.

### Assessment of Cognitive Functions

Cognitive testing was performed using a computerized tablet-based EF assessment battery (Lussier et al., 2020), which includes a digit Stroop task (allowing us to examine the EF inhibition, global and local switching subcomponents) and an n-back task (updating). Before each task, detailed instructions appeared on the screen (which were also read aloud), followed by a familiarization block.

**FIGURE 1:**
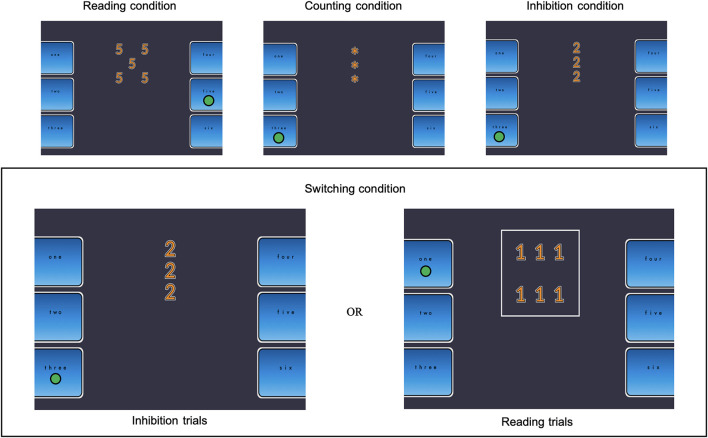
Illustration of the Stroop task condition. The green circle represents the correct response for each condition trail.

The digit Stroop task was modeled after the Five Digit test (de Paula et al., 2017); a digit-Stroop. The Stroop task consisted of four distinct conditions: reading (60 trials), counting (60 trials), inhibition (60 trials), and switching (73 non-switching trials and 48 switching trials). Figure 1 illustrates the Stroop task conditions. Participants were instructed to respond as quickly as possible while minimizing errors by pressing the buttons on either the right or left side of the screen with their thumbs. In the reading condition, participants had to identify the digit (from 1 to 6) displayed on the screen. Up to six digits could be presented simultaneously on the screen. The digits and the quantity of digits were always matched (e.g., digit 2 appearing at two locations). In the counting condition, participants had to report the number of asterisks (up to six asterisks could be presented). In the inhibition condition, participants had to report how many digits (up to six) appeared on the screen. In this condition, the identity of the digits presented on screen was incompatible with the quantity of digits displayed (e.g., the digit “4” appearing at five locations on the screen). In the switching condition, the instructions were the same as in the inhibition condition, except for the trials (i.e., 48 switching trials out of the 121 trials) in which the digits were surrounded by a white frame, indicating that participants had to report the identity of the digit instead of the number of digits presented. For all conditions, two consecutive trials never gave the same answer. Also, the number used as an answer was equiprobable.

**FIGURE 2:**
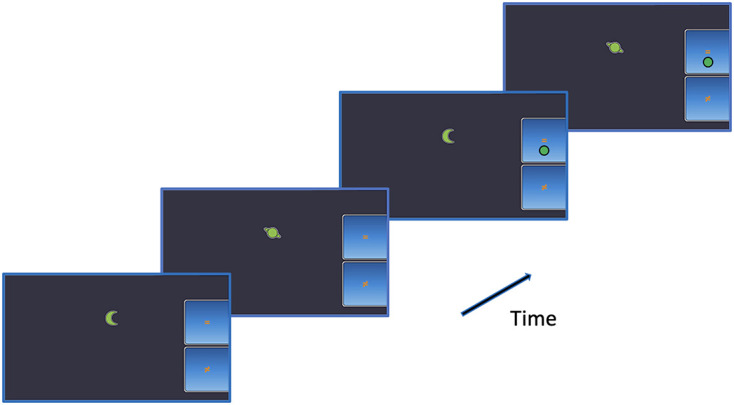
Illustration of the 2-back task. The green circle represents the correct response.

The n-back task included three conditions: 1-back (4 blocks of 10 trials), 2-back (4 blocks of 10 trials), and 3-back (2 blocks of 10 trials). Figure 2 illustrates some of the trials of the 2-back condition. Participants were asked to indicate whether a non-verbal stimulus displayed on the screen was identical (by pressing the “=” button) or different (by pressing the “≠” button) to the one presented *n* steps earlier in the sequence. The participant had to use his right thumb to enter his answer. Participants were asked to be as accurate as possible and to respond within a 3,000 ms delay. Each block consisted of 40% target and 60% non-target letters. The duration of the inter-stimulus interval was 750 ms. During this period, accuracy feedback was presented based on the participant’s last response, such that the pressed button turned green (accurate answer) or red (error). If the participant accuracy was 75% or more in one condition, the following n-back condition was activated. If not, the task ended. Only the performance on the 1-back and the 2-back were analyzed because most participants were unable to perform the 3-back.

Performance on each EF was computed in terms of different costs calculated as a percentage (interference, local switch, global switch, and updating cost) using reaction time (RT). When calculating costs, it is common to compare the more complex condition to a simpler condition to isolate the process that differentiates them. Therefore, to isolate the local switching cost (the cost associated with alternating between two tasks), we used the performance of the switching trials and the non-switching trials. To isolate the global switching cost, the cost of maintaining and coordinating two tasks relative to performing a single task, we used the performance of the non-switching trials and the inhibition trials. The rationale is that both trials share the same visual presentation and instruction, except that in the non-switching trials the participant has to keep in mind two sets of instruction and prepare themselves accordingly as they don’t know in advance which trials (non-switching trials vs. switching trials) will be presented [task uncertainty (Kray et al., 2002)]. To isolate the updating cost, we used the respective performance on the 2-back (which is demanding in terms of updating) and the performance of the 1-back (which barely necessitates updating).

The costs were computed according to standard procedures (Li et al., 2018). *Interference cost*: [(inhibition trial RT – counting trial RT) / counting trial RT], *local switching cost*: (switching trial RT – non-switching trial RT) / non-switching trial RT, *global switching cost*: (non-switching trial RT – inhibition trial RT) / inhibition trial RT, and *updating cost*: (n-back 2 trial RT – n-back 1 trial RT) / n-back 1 trial RT. Only the RTs associated with a correct response were included in the computation of the respective cost. Maximum RT of 4,000 ms and a minimum RT of 200 ms were allowed. Table 1 shows participants’ cognitive data.

### Statistical Analysis

The normality assumption was tested using the Kolmogorov–Smirnov test and the Shapiro–Wilk test. The CRFe on the ergocycle was determined as the reference CRFe in the agreement analysis. Systematic bias was assessed using a paired *t*-test. The magnitude of the difference (Cohen, 1988) was classified using the effect size approach: small (0.2), moderate (0.5), and large (0.8). A regression analysis of the differences was computed to assess proportional bias (Bland and Altman, 1999). Pearson’s correlation coefficients (r) were also computed to assess linear relationship between each estimate. The strength of the associations was interpreted as very weak (0–0.19), weak (0.2–0.39), moderate (0.40–0.59), strong (0.6–0.79), and very strong (0.8–1) (Campbell and Swinscow, 2009). Fisher r-to-z transformations were calculated to assess whether there were significant differences between the correlation coefficients.

To assess the relationship between CRFe and EF, two-stage multiple hierarchical regression analyses were performed. Age and education were entered in the first block. In addition to age and education, CRF scores (1 analysis for each estimate) were entered in the second block. Dependent variables were the i*nterference cost*, the l*ocal switching cost*, the *global switching cost*, and the *updating cost*. All analyses were performed using Statistical Package for the Social Sciences 27.0 (SPSS Inc., Chicago, IL, United States) and statistical significance was set at *p* < 0.05.

## Results

### Cardiorespiratory Fitness Estimates Agreement Analysis

On the basis of the RER criterion (≥1.1), seven participants were excluded from GXT-specific analyses. Seven participants (including one overlapping with the RER criterion) were also excluded from the ROCKPORT-specific analyses because their CRFe were deemed to be too low. These participants’ CRFe values were below a cutoff (10.8 ml.kg^–1^.min^–1^) determined by previously collected $\dot{V}\,O_{2}$ peak data from a GXT performed on a cycle ergometer in a similar sample of 59 healthy older adult participants.

In terms of systematic bias, a moderate difference (*d* = 0.58) was found between GXT (21.61 ml.kg^–1^.min^–1^ ± 4.56) and EQUATION (24.59 ml.kg^–1^.min^–1^ ± 6.63); *t*(52) = −4.26, *p* < 0.01. A moderate significant difference (*d* = 0.51) was also found between GXT (22.35 ml.kg^–1^.min^–1^ ± 4.25) and ROCKPORT (20.37 ml.kg^–1^.min^–1^ ± 6.03); *t*(46) = 2.75, *p* < 0.01. A large significant difference (*d* = −0.91) was also noted between EQUATION (25.22 ml.kg^–1^.min^–1^ ± 6.38) and ROCKPORT (20.04 ml.kg^–1^.min^–1^ ± 5.84); *t*(52) = 6.26, *p* < 0.01. Based on the *p*-value of regression coefficients, no proportional bias was observed, suggesting that the differences between the methods were similar across all the CRF levels.

A strong positive correlation was found between GXT and EQUATION; *r*(51) = 0.64, *p* < 0.01. A moderate positive correlation was observed between GXT and ROCKPORT; *r*(45) = 0.58, *p* < 0.01, and the EQUATION and the ROCKPORT, *r*(51) = 0.52, *p* < 0.01. Using Fisher r-to-z transformation, no difference was found between correlation coefficients.

### Relation Between Cardiorespiratory Fitness Estimates and Executive Function

Because the analysis included fewer than five independent variables, a sample size of 60 was considered adequate (Harris, 1985). The assumption of singularity was met, as no independent variable correlated with another at 0.70 or greater. Hierarchical regressions were calculated to predict EF costs based on Age and Education (Model 1) and Age, Education, and a CRFe (Model 2). Note that Age is included in the computation of ROCKPORT and EQUATION. Significant associations were observed between Age and GXT (−0.32) and Age and ROCKPORT (−0.47), whereas no significant association was found between Age and the EQUATION. Tolerance (>0.2) and VIF (<10) were all within acceptable limits. Mahalanobis distance scores indicated no multivariate outliers. Table 2 presents the summary results of the hierarchical regressions.

**TABLE 2:** Summary of regression analyses predicting executive function performance.

	*R* ^2^	*ΔR^2^*	*ΔF*		*R* ^2^	*ΔR^2^*	*ΔF*

Global switching cost				Interference cost			
Model 1	0.014	0.014	0.401	Model 1	0.079	0.079	2.456
Model 2 (GXT)	0.129	0.115	7.425*	Model 2 (GXT)	0.170	0.091	6.108*
Model 1	0.014	0.014	0.401	Model 1	0.079	0.079	2.456
Model 2 (ROCK)	0.164	0.150	10.048*	Model 2 (ROCK)	0.158	0.079	5.240*
Model 1	0.014	0.014	0.401	Model 1	0.079	0.079	2.456
Model 2 (EQUA)	0.040	0.026	1.500	Model 2 (EQUA)	0.179	0.100	6.822*

**Local switching cost**				**Updating cost**			

Model 1	0.001	0.001	0.033	Model 1	0.015	0.015	0.434
Model 2 (GXT)	0.002	0.000	0.022	Model 2 (GXT)	0.019	0.004	0.197
Model 1	0.001	0.001	0.033	Model 1	0.015	0.015	0.434
Model 2 (ROCK)	0.020	0.019	1.079	Model 2 (ROCK)	0.025	0.010	0.548
Model 1	0.001	0.001	0.033	Model 1	0.015	0.015	0.434
Model 2 (EQUA)	0.003	0.001	0.084	Model 2 (EQUA)	0.032	0.016	0.930

*Model 1, Age and education; Model 2, Age and education and cardiorespiratory fitness estimate.*

*p < 0.05.

*GXT, Maximal graded exercise test; EQUA, Non-exercise prediction equation; ROCK, Rockport Fitness Walking Test.*

**FIGURE 3:**
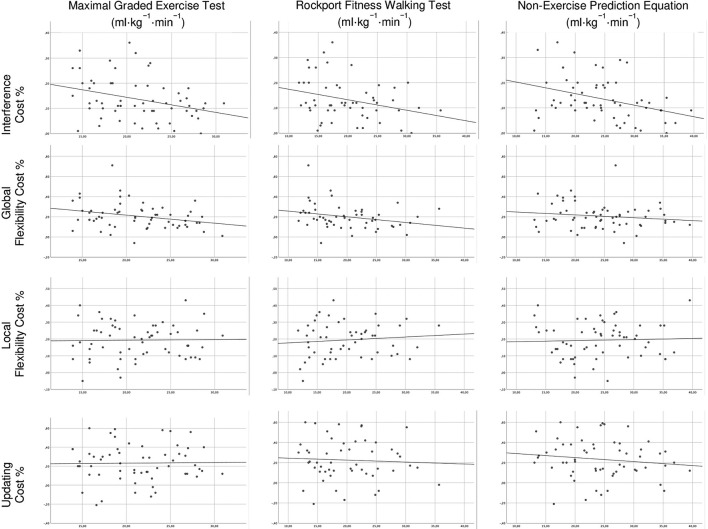
Scatter plots of the associations between cardiorespiratory fitness estimates and executive functions costs.

For each cost, no significant regression was found with Model 1. For the *global switching cost*, Model 2 with GXT, ROCKPORT or EQUATION, explained 13% (*p* < 0.01), 16% (*p* < 0.01), and 4% (*p* = ns) of the variance, respectively. For the *interference cost*, Model 2 with GXT, ROCKPORT or EQUATION explained 17% (*p* < 0.05), 16% (*p* < 0.05), and 18% (*p* < 0.05) of the variance, respectively. For the *local switching cost* and the *updating cost*, no significant regression was found for Model 2, regardless of the CRFe included. Figure 3 represents the scatter plots of the associations between CRFe and EF costs.

Table 3 shows the regression coefficients. In Model 2 with GXT, CRF (β = −0.361, *p* < 0.01) was significantly related to *global switching cost*. In addition, GXT (β = −0.320, *p* < 0.05) and Education (β = −0.261, *p* < 0.05) were significant predictors of the *interference cost*. In Model 2 with ROCKPORT, CRF (β = −0.442, *p* < 0.01) and Age (β = −0.326, *p* < 0.05) were significant predictors of the *global switching cost*. Also, ROCKPORT (β = −0.321, *p* < 0.05) and Education (β = −0.209, *p* < 0.05) were significant predictors of the *interference cost*. For Model 2 with EQUATION, CRF (β = −0.321, *p* < 0.05) was a significant predictor of the *interference cost*.

**TABLE 3:** Regression coefficients of the demographic and cardiorespiratory fitness predictors of executive function performance.

	β	*B*	*p*		β	*B*	*p*

Global switching cost				Interference cost			
Age	–0.233	−0.005	0.090	Age	0.170	0.000	0.897
Education	–0.113	−0.004	0.382	Education	–0.261	–0.006	0.042*
GXT	–0.361	−0.010	0.009*	GXT	–0.320	–0.006	0.017*
Age	–0.326	−0.008	0.026*	Age	–0.030	0.000	0.834
Education	–0.115	−0.004	0.365	Education	–0.256	–0.006	0.047*
ROCK	–0.442	−0.007	0.002*	ROCK	–0.321	–0.004	0.026*
Age	–0.132	−0.003	0.332	Age	0.080	0.001	0.522
Education	–0.065	−0.002	0.628	Education	–0.209	–0.005	0.096
EQUA	–0.163	−0.003	0.225	EQUA	–0.321	–0.004	0.012*

**Local switching cost**				**Updating cost**			

Age	–0.012	0.000	0.936	Age	0.127	0.004	0.387
Education	0.027	0.001	0.844	Education	0.098	0.005	0.479
GXT	0.021	0.000	0.883	GXT	0.063	0.003	0.659
Age	0.058	0.001	0.706	Age	0.048	0.002	0.756
Education	0.039	0.001	0.773	Education	0.079	0.004	0.567
ROCK	0.157	0.002	0.303	ROCK	–0.113	–0.003	0.462
Age	–0.013	0.000	0.924	Age	0.085	0.003	0.536
Education	0.023	0.001	0.868	Education	0.098	0.005	0.472
EQUA	0.039	0.001	0.774	EQUA	–0.130	–0.004	0.339

*GXT, Maximal graded exercise test; EQUA, Non-exercise prediction equation; ROCK, Rockport Fitness Walking Test.*

*p < 0.05.

## Discussion

The purpose of this study was to examine the associations between three CRFe (GXT, ROCKPORT, EQUATION) and EF (switching, inhibition, and working-memory updating) in healthy older adults. All CRFe were associated with the *interference cost*. We found significant negative relationships between the *global switching cost* and the GXT as well as the ROCKPORT. This suggests that exercise-based protocols, such as GXT and ROCKPORT, appear to be better predictors of the relationships between CRF and EF, as they yielded consistent results for the different EF subcomponents. While the variances explained by each CRFe are statistically significant, they remain small and should be interpreted with caution in terms of clinical significance. No significant relation was observed between the *global switching cost* and EQUATION. Such a discrepancy could stem from the inherent challenge of self-reporting physical activity levels (Sylvia et al., 2014), a variable included in the EQUATION. No relationship was observed between the three CRFe and the *local switching costs* or the *updating costs*. It has been reported that global switching is more sensitive to age-related decline compared to local switching (Verhaeghen and Cerella, 2002; Wasylyshyn et al., 2011; Ferguson et al., 2021). Therefore, it is possible that similarly to updating, local switching is less sensitive to age-related decline in CRF.

Although our study highlighted the diversity of EFs, as not all EFs subcomponents are related to CRFe, we cannot reject the possibility that the associations between GXT and ROCKPORT CRFe and the *global switching* and *interference cost*, are related to shared cognitive processes. This would be compatible with the proposal that the inhibition function depends on more fundamental processes (i.e., goal maintenance and management) that are shared with other EFs (Friedman et al., 2008, 2011), and in the case of the present study, particularly with global switching.

Correlation coefficients between the three CRFe (characterized as moderate to strong) were similar to previous studies (Mailey et al., 2010; McAuley et al., 2011), suggesting that the three CRFe are equivalent. However, in our study, we detect the presence of a systematic bias. The EQUATION and the ROCKPORT estimates, respectively, overestimated and underestimated the GXT score. Previous studies have also reported overestimation of CRF by the EQUATION (McAuley et al., 2011) and underestimation of CRF by the ROCKPORT (Fenstermaker et al., 1992; Warren et al., 1993) when compared to a GXT performed on a treadmill. Despite these differences in agreement between each CRFe, similar negative associations were found between each CRFe and *interference cost*. Our findings also suggest that education is a significant predictor of *interference cost* in the model that included GXT or ROCKPORT. Further research in older adults is needed to explain how educational attainment (regardless of the effect of age) might affect a specific cognitive domain (Boller et al., 2017).

Based on our results, CRFe explains a small percentage of variance in EF performance in older adults. Further research is needed to explain how this occurs and how CRF interacts with other mechanisms associated with brain health. For example, the cerebral circulation hypothesis (Rogers et al., 1990) suggests a better vascularization of the frontal lobes. This would benefit performance in tasks requiring EF, as they depend on the integrity of the subcortical frontal circuit, which includes the prefrontal cortex (Gunning-Dixon and Raz, 2003; Alvarez and Emory, 2006). The hypothesis of neurotrophic stimulation (Spirduso, 1980) proposed that the benefit could be related to the production of growth factors, such as Brain-derived neurotrophic factor (BDNF; Szuhany et al., 2015) and Insulin-like growth factor 1 (IGF-1; Cheng et al., 2000) that affect brain metabolism. Other hypotheses also point to preserved vessel elasticity (Gauthier et al., 2015), highlight the importance of inflammation-related markers (Cotman et al., 2007) and their impacts namely on sarcopenia and muscle strength (Schaap et al., 2006), or posit that dopaminergic neurotransmission (Backman et al., 2010) possibly changes after physical exercise (Petzinger et al., 2015).

An important limitation of this study is the small sample size, which limits the number of predictors we could use. Also, because the sample was homogeneous, the potential effect of biological sex could not be analyzed. Furthermore, the cross-sectional design does not allow conclusions to be drawn about the causality of the relationship between CRFe and EF. Another limitation of our study is that the use of beta blockers, potentially related to a reduction in CRF (Nielen et al., 2019), was not evaluated.

One of the strengths of the present study lies in the detailed analyses of the respective EF. From the creation of the tasks to the cost computation, initiatives were taken to isolate age-related declines in EF from a more general decline in processing speed. The use of a computerized assessment may be more sensitive to detect differences in EF in a sample of healthy older adults than traditional neuropsychological assessment (Erickson et al., 2019). This study is innovative in measuring three different CRFe in the same sample of older adults, including a GXT performed on a cycle ergometer. Estimation of CRF during a GXT performed on a cycle ergometer has been reported in numerous intervention studies (Blumenthal et al., 1991; Fabre et al., 2002; Jonasson et al., 2016) and offers many advantages in terms of cost, space, portability and ease of simultaneous measurement of other physiological measures (Smith et al., 2016). Indeed, a test without weight-bearing (Huggett et al., 2005) that does not depend upon the participants’ walking ability, especially on a treadmill, might be easier to implement in frail older adults, populations with balance issues, increased risks of falls, orthopedic restrictions (Beltz et al., 2016), joint pain or poor lower limb control (e.g., after a stroke) (Tang et al., 2013).

This study expands our knowledge about the relationship between CRFe and cognitive functions. We examined whether different CRFe were similarly associated with each EF subcomponent in healthy older adults. Comparable relations were observed between the GXT and ROCKPORT estimates with each EF subcomponents. The absence of a relation between the EQUATION and global switch cost indicates that exercise-based protocols might be more suitable in assessing the relationship between CRF and cognition in older adults The EF subcomponent that was associated with CRFe as measured by the GXT and ROCKPORT, are those known to be the most affected by aging (Maldonado et al., 2020). In their meta-analysis of cross-sectional studies, Maldonado et al. (2020) primarily computed EF performance in terms of RTs or accuracies for a single task, which may limit their ability to isolate EF performance from a more general age-related change in processing speed. Although the use of a difference score computation used in the present study may reduce the likelihood that these associations reflect age differences in processing speed, it cannot exclude it. Therefore, our results should also be interpreted with caution.

Aging is a multifactorial process, however, growing evidence shows that age-related changes in EF performance are related to changes in the integrity of several brain regions (Milham et al., 2002; Jolly et al., 2017), including the prefrontal cortex (Allen et al., 2005; Lemaitre et al., 2005; Coubard et al., 2011). In cross-sectional studies, higher CRF was associated with larger hippocampal volume (Erickson et al., 2009) and had a protective effect on white matter integrity (Marks et al., 2007; Johnson et al., 2012). As CRF is a modifiable factor, improving CRF through the adoption of an active lifestyle could possibly mitigate the brain from age-related insult. The same conclusion could be applied to a reduction of cerebrovascular risk factors. In a recent study, Veldsman et al. (2020) report that in older adults, cerebrovascular risk factors were associated with reduced integrity of the frontoparietal network that affects EF.

Future studies assessing the associations between CRFe and EF subcomponents (particularly inhibition and global switching costs as assessed in the present study) could benefit from the use of neuroimaging techniques such as fMRI (Nashiro et al., 2018), functional near-infrared spectroscopy (Lague-Beauvais et al., 2013; Mekari et al., 2019) and electroencephalography (Gajewski and Falkenstein, 2015b; Gajewski et al., 2018). We also encourage investigators to examine the association between cognitive function (particularly EF) and CRFe in clinical populations, such as people living with mild cognitive impairment (Stuckenschneider et al., 2018), as they are known to present global switching impairment (Belleville et al., 2008). The present results support the promotion of the use of exercise-based protocols to assess CRF when possible and the inclusion of at least one task per EF subcomponents. We also recommended the standardization of the cognitive test batteries used (Voss et al., 2019), since it would improve the comparability between studies from different research groups.

## Data Availability Statement

The data from this study, including anonymized participant-level and study-level data (analyzable data sets) and other information (such as protocols), will be shared with qualified researchers as necessary for conducting legitimate research through a direct request from the study principal investigator (LB at louis.bherer@umontreal.ca).

## Ethics Statement

The participants provided their written informed consent to participate in this study. The study protocol was approved by the Research Ethics Board of the Institut Universitaire de Gériatrie de Montréal (IUGM). The study was part of a larger clinical trial (NCT02455258).

## Author Contributions

LB conceived the grant application to the Quebec Ministry of Health and Social Services. DP contributed to the study design and provided the first draft of the manuscript. ML, NB, and LB actively contributed to manuscript writing and statistical analyses. DP, NB, FC, TV, and JV tested the participants. All authors played a specific role in the study based on their expertise, either by providing advice, measurement tools, and expertise.

## Conflict of Interest

The authors declare that the research was conducted in the absence of any commercial or financial relationships that could be construed as a potential conflict of interest.

## Publisher’s Note

All claims expressed in this article are solely those of the authors and do not necessarily represent those of their affiliated organizations, or those of the publisher, the editors and the reviewers. Any product that may be evaluated in this article, or claim that may be made by its manufacturer, is not guaranteed or endorsed by the publisher.

## References

[B1] Albinet C. T. Boucard G. Bouquet C. A. Audiffren M. (2012). Processing speed and executive functions in cognitive aging: how to disentangle their mutual relationship? *Brain Cogn.* 79 1–11. 10.1016/j.bandc.2012.02.001 22387275

[B2] Albinet C. T. Mandrick K. Bernard P. L. Perrey S. Blain H. (2014). Improved cerebral oxygenation response and executive performance as a function of cardiorespiratory fitness in older women: a fNIRS study. *Front. Aging Neurosci.* 6:272. 10.3389/fnagi.2014.00272 25339900 PMC4189417

[B3] Allen J. S. Bruss J. Brown C. K. Damasio H. (2005). Normal neuroanatomical variation due to age: the major lobes and a parcellation of the temporal region. *Neurobiol. Aging* 26 1245–1260; discussion 1279–1282. 10.1016/j.neurobiolaging.2005.05.023 16046030

[B4] Alvarez J. A. Emory E. (2006). Executive function and the frontal lobes: a meta-analytic review. *Neuropsychol. Rev.* 16 17–42. 10.1007/s11065-006-9002-x 16794878

[B5] Anstey K. J. Wood J. Lord S. Walker J. G. (2005). Cognitive, sensory and physical factors enabling driving safety in older adults. *Clin. Psychol. Rev.* 25 45–65. 10.1016/j.cpr.2004.07.008 15596080

[B6] Backman L. Lindenberger U. Li S. C. Nyberg L. (2010). Linking cognitive aging to alterations in dopamine neurotransmitter functioning: recent data and future avenues. *Neurosci. Biobehav. Rev.* 34 670–677. 10.1016/j.neubiorev.2009.12.008 20026186

[B7] Balady G. J. Arena R. Sietsema K. Myers J. Coke L. Fletcher G. F. (2010). Clinician’s Guide to cardiopulmonary exercise testing in adults: a scientific statement from the American Heart Association. *Circulation* 122 191–225. 10.1161/CIR.0b013e3181e52e69 20585013

[B8] Banich M. T. (2009). Executive functionthe search for an integrated account. *Curr. Dir. Psychol. Sci.* 18 89–94. 10.1111/j.1467-8721.2009.01615.x

[B9] Barnes D. E. Yaffe K. Satariano W. A. Tager I. B. (2003). A longitudinal study of cardiorespiratory fitness and cognitive function in healthy older adults. *J. Am. Geriatr. Soc.* 51 459–465. 12657064 10.1046/j.1532-5415.2003.51153.x

[B10] Belleville S. Bherer L. Lepage E. Chertkow H. Gauthier S. (2008). Task switching capacities in persons with Alzheimer’s disease and mild cognitive impairment. *Neuropsychologia* 46 2225–2233. 10.1016/j.neuropsychologia.2008.02.012 18374374

[B11] Beltz N. M. Gibson A. L. Janot J. M. Kravitz L. Mermier C. M. Dalleck L. C. (2016). Graded exercise testing protocols for the determination of VO2max: historical perspectives, progress, and future considerations. *J. Sports Med. (Hindawi Publ. Corp.)* 2016:3968393. 10.1155/2016/3968393 28116349 PMC5221270

[B12] Berryman N. Bherer L. Nadeau S. Lauziere S. Lehr L. Bobeuf F. (2013). Executive functions, physical fitness and mobility in well-functioning older adults. *Exp. Gerontol.* 48 1402–1409. 10.1016/j.exger.2013.08.017 24012563

[B13] Berryman N. Bherer L. Nadeau S. Lauziere S. Lehr L. Bobeuf F. (2014). Multiple roads lead to Rome: combined high-intensity aerobic and strength training vs. gross motor activities leads to equivalent improvement in executive functions in a cohort of healthy older adults. *Age (Dordr)* 36:9710. 10.1007/s11357-014-9710-8 25194940 PMC4156938

[B14] Berryman N. Pothier K. Bherer L. (2018). “Physical activity, exercise and executive functions,” in *Executive Function: Development Across The Life Span*, eds Wiebe S. Karbach J. (New York, NY: Taylor & Francis).

[B15] Bland J. M. Altman D. G. (1999). Measuring agreement in method comparison studies. *Stat. Methods Med. Res.* 8 135–160. 10.1177/096228029900800204 10501650

[B16] Blumenthal J. A. Emery C. F. Madden D. J. Schniebolk S. Walsh-Riddle M. George L. K. (1991). Long-term effects of exercise on psychological functioning in older men and women. *J. Gerontol.* 46 352–361. 10.1093/geronj/46.6.p352 1940092

[B17] Boller B. Mellah S. Ducharme-Laliberte G. Belleville S. (2017). Relationships between years of education, regional grey matter volumes, and working memory-related brain activity in healthy older adults. *Brain Imaging Behav.* 11 304–317. 10.1007/s11682-016-9621-7 27734304

[B18] Boucard G. K. Albinet C. T. Bugaiska A. Bouquet C. A. Clarys D. Audiffren M. (2012). Impact of physical activity on executive functions in aging: a selective effect on inhibition among old adults. *J. Sport Exerc. Psychol.* 34 808–827. 23204360 10.1123/jsep.34.6.808

[B19] Brown A. D. McMorris C. A. Longman R. S. Leigh R. Hill M. D. Friedenreich C. M. (2010). Effects of cardiorespiratory fitness and cerebral blood flow on cognitive outcomes in older women. *Neurobiol. Aging* 31 2047–2057. 10.1016/j.neurobiolaging.2008.11.002 19111937

[B20] Cahn-Weiner D. A. Boyle P. A. Malloy P. F. (2002). Tests of executive function predict instrumental activities of daily living in community-dwelling older individuals. *Appl. Neuropsychol.* 9 187–191. 10.1207/S15324826AN0903_8 12584085

[B21] Campbell M. J. Swinscow T. D. V. (2009). *Statistics At Square One.* Hoboken, NJ: Wiley-Blackwell.

[B22] Cheng C. M. Reinhardt R. R. Lee W. H. Joncas G. Patel S. C. Bondy C. A. (2000). Insulin-like growth factor 1 regulates developing brain glucose metabolism. *Proc. Natl. Acad. Sci. U.S.A.* 97 10236–10241. 10.1073/pnas.170008497 10954733 PMC27834

[B23] Cohen J. (1988). *Statistical Power Analysis For The Behavioral Sciences.* Hillsdale, NJ: L. Erlbaum Associates.

[B24] Colcombe S. J. Kramer A. F. Erickson K. I. Scalf P. McAuley E. Cohen N. J. (2004). Cardiovascular fitness, cortical plasticity, and aging. *Proc. Natl. Acad. Sci. U.S.A.* 101 3316–3321. 10.1073/pnas.0400266101 14978288 PMC373255

[B25] Cotman C. W. Berchtold N. C. Christie L. A. (2007). Exercise builds brain health: key roles of growth factor cascades and inflammation. *Trends Neurosci.* 30 464–472. 10.1016/j.tins.2007.06.011 17765329

[B26] Coubard O. A. Ferrufino L. Boura M. Gripon A. Renaud M. Bherer L. (2011). Attentional control in normal aging and Alzheimer’s disease. *Neuropsychology* 25 353–367. 10.1037/a0022058 21417533

[B27] Davis J. C. Marra C. A. Najafzadeh M. Liu-Ambrose T. (2010). The independent contribution of executive functions to health related quality of life in older women. *BMC Geriatr.* 10:16. 10.1186/1471-2318-10-16 20359355 PMC2867806

[B28] de Fockert J. W. Rees G. Frith C. D. Lavie N. (2001). The role of working memory in visual selective attention. *Science* 291 1803–1806. 10.1126/science.1056496 11230699

[B29] de Paula J. J. Oliveira T. D. O. Querino E. H. G. Malloy-Diniz L. F. (2017). The Five Digits Test in the assessment of older adults with low formal education: construct validity and reliability in a Brazilian clinical sample. *Trends Psychiatry Psychother.* 39 173–179. 28876361 10.1590/2237-6089-2016-0060

[B30] Erickson K. I. Hillman C. Stillman C. M. Ballard R. M. Bloodgood B. Conroy D. E. (2019). Physical activity, cognition, and brain outcomes: a review of the 2018 physical activity guidelines. *Med. Sci. Sports Exerc.* 51 1242–1251. 10.1249/MSS.0000000000001936 31095081 PMC6527141

[B31] Erickson K. I. Prakash R. S. Voss M. W. Chaddock L. Hu L. Morris K. S. (2009). Aerobic fitness is associated with hippocampal volume in elderly humans. *Hippocampus* 19 1030–1039. 10.1002/hipo.20547 19123237 PMC3072565

[B32] Fabre C. Chamari K. Mucci P. Masse-Biron J. Prefaut C. (2002). Improvement of cognitive function by mental and/or individualized aerobic training in healthy elderly subjects. *Int. J. Sports Med.* 23 415–421. 10.1055/s-2002-33735 12215960

[B33] Fenstermaker K. L. Plowman S. A. Looney M. A. (1992). Validation of the rockport fitness walking test in females 65 years and older. *Res. Q. Exerc. Sport* 63 322–327. 10.1080/02701367.1992.10608749 1513964

[B34] Ferguson H. J. Brunsdon V. E. A. Bradford E. E. F. (2021). The developmental trajectories of executive function from adolescence to old age. *Sci. Rep.* 11:1382. 10.1038/s41598-020-80866-1 33446798 PMC7809200

[B35] Folstein M. F. Folstein S. E. McHugh P. R. (1975). “Mini-mental state”. A practical method for grading the cognitive state of patients for the clinician. *J. Psychiatr. Res.* 12 189–198. 1202204 10.1016/0022-3956(75)90026-6

[B36] Freudenberger P. Petrovic K. Sen A. Toglhofer A. M. Fixa A. Hofer E. (2016). Fitness and cognition in the elderly: the austrian stroke prevention study. *Neurology* 86 418–424. 10.1212/WNL.0000000000002329 26740674 PMC4773949

[B37] Friedman N. P. Miyake A. (2017). Unity and diversity of executive functions: individual differences as a window on cognitive structure. *Cortex* 86 186–204. 10.1016/j.cortex.2016.04.023 27251123 PMC5104682

[B38] Friedman N. P. Miyake A. Robinson J. L. Hewitt J. K. (2011). Developmental trajectories in toddlers’ self-restraint predict individual differences in executive functions 14 years later: a behavioral genetic analysis. *Dev. Psychol.* 47 1410–1430. 10.1037/a0023750 21668099 PMC3168720

[B39] Friedman N. P. Miyake A. Young S. E. DeFries J. C. Corley R. P. Hewitt J. K. (2008). Individual differences in executive functions are almost entirely genetic in origin. *J. Exp. Psychol. Gen.* 137 201–225. 10.1037/0096-3445.137.2.201 18473654 PMC2762790

[B40] Gajewski P. D. Falkenstein M. (2015a). Lifelong physical activity and executive functions in older age assessed by memory based task switching. *Neuropsychologia* 73 195–207. 10.1016/j.neuropsychologia.2015.04.031 25937323

[B41] Gajewski P. D. Falkenstein M. (2015b). Long-term habitual physical activity is associated with lower distractibility in a Stroop interference task in aging: behavioral and ERP evidence. *Brain Cogn.* 98 87–101. 10.1016/j.bandc.2015.06.004 26160263

[B42] Gajewski P. D. Ferdinand N. K. Kray J. Falkenstein M. (2018). Understanding sources of adult age differences in task switching: evidence from behavioral and ERP studies. *Neurosci. Biobehav. Rev.* 92 255–275. 10.1016/j.neubiorev.2018.05.029 29885425

[B43] Gauthier C. J. Lefort M. Mekary S. Desjardins-Crepeau L. Skimminge A. Iversen P. (2015). Hearts and minds: linking vascular rigidity and aerobic fitness with cognitive aging. *Neurobiol. Aging* 36 304–314. 10.1016/j.neurobiolaging.2014.08.018 25308963

[B44] Gunning-Dixon F. M. Raz N. (2003). Neuroanatomical correlates of selected executive functions in middle-aged and older adults: a prospective MRI study. *Neuropsychologia* 41 1929–1941. 14572526 10.1016/s0028-3932(03)00129-5

[B45] Harris R. J. (1985). *A primer Of Multivariate Statistics.* Orlando: Academic Press.

[B46] Hawkins S. Wiswell R. (2003). Rate and mechanism of maximal oxygen consumption decline with aging: implications for exercise training. *Sports Med.* 33 877–888. 10.2165/00007256-200333120-00002 12974656

[B47] Hayes S. M. Forman D. E. Verfaellie M. (2016). Cardiorespiratory fitness is associated with cognitive performance in older but not younger adults. *J. Gerontol. B Psychol. Sci. Soc. Sci.* 71 474–482. 10.1093/geronb/gbu167 25528256 PMC6959383

[B48] Hayes S. M. Hayes J. P. Cadden M. Verfaellie M. (2013). A review of cardiorespiratory fitness-related neuroplasticity in the aging brain. *Front. Aging Neurosci.* 5:31. 10.3389/fnagi.2013.00031 23874299 PMC3709413

[B49] Huggett D. L. Connelly D. M. Overend T. J. (2005). Maximal aerobic capacity testing of older adults: a critical review. *J. Gerontol. A Biol. Sci. Med. Sci.* 60 57–66. 10.1093/gerona/60.1.57 15741284

[B50] Insel K. Morrow D. Brewer B. Figueredo A. (2006). Executive function, working memory, and medication adherence among older adults. *J. Gerontol. B Psychol. Sci. Soc. Sci.* 61 102–107. 10.1093/geronb/61.2.p102 16497953

[B51] Johnson N. F. Kim C. Clasey J. L. Bailey A. Gold B. T. (2012). Cardiorespiratory fitness is positively correlated with cerebral white matter integrity in healthy seniors. *Neuroimage* 59 1514–1523. 10.1016/j.neuroimage.2011.08.032 21875674 PMC3230672

[B52] Jolly T. A. Cooper P. S. Rennie J. L. Levi C. R. Lenroot R. Parsons M. W. (2017). Age-related decline in task switching is linked to both global and tract-specific changes in white matter microstructure. *Hum. Brain Mapp.* 38 1588–1603. 10.1002/hbm.23473 27879030 PMC6866847

[B53] Jonasson L. S. Nyberg L. Kramer A. F. Lundquist A. Riklund K. Boraxbekk C. J. (2016). Aerobic exercise intervention, cognitive performance, and brain structure: results from the physical influences on brain in aging (PHIBRA) study. *Front. Aging Neurosci.* 8:336. 10.3389/fnagi.2016.00336 28149277 PMC5241294

[B54] Jurca R. Jackson A. S. LaMonte M. J. Morrow J. R. Jr. Blair S. N. Wareham N. J. (2005). Assessing cardiorespiratory fitness without performing exercise testing. *Am. J. Prev. Med.* 29 185–193. 10.1016/j.amepre.2005.06.004 16168867

[B55] Klein R. (1996). Attention: selection, awareness, and control – a tribute to Donald Broadbent. *Am. J. Psychol.* 109 139–150.

[B56] Kline G. M. Porcari J. P. Hintermeister R. Freedson P. S. Ward A. McCarron R. F. (1987). Estimation of VO2max from a one-mile track walk, gender, age, and body weight. *Med. Sci. Sports Exerc.* 19 253–259. 3600239

[B57] Kray J. Dörrenbächer S. (2019). “The effectiveness of training in task switching: new insights and open issues from a life-span view,” in *Cognitive And Working Memory Training: Perspectives From Psychology, Neuroscience, And Human Development*, eds Novick J. M. Bunting M. F. Dougherty M. R. Engle R. W. (New York, NY: Oxford University Press), 508–538.

[B58] Kray J. Lindenberger U. (2000). Adult age differences in task switching. *Psychol. Aging* 15 126–147. 10755295 10.1037//0882-7974.15.1.126

[B59] Kray J. Li K. Z. Lindenberger U. (2002). Age-related changes in task-switching components: the role of task uncertainty. *Brain Cogn.* 49 363–381. 10.1006/brcg.2001.1505 12139959

[B60] Lague-Beauvais M. Brunet J. Gagnon L. Lesage F. Bherer L. (2013). A fNIRS investigation of switching and inhibition during the modified Stroop task in younger and older adults. *Neuroimage* 64 485–495. 10.1016/j.neuroimage.2012.09.042 23000257

[B61] Larson E. B. Wang L. Bowen J. D. McCormick W. C. Teri L. Crane P. (2006). Exercise is associated with reduced risk for incident dementia among persons 65 years of age and older. *Ann. Intern. Med.* 144 73–81. 16418406 10.7326/0003-4819-144-2-200601170-00004

[B62] Lemaitre H. Crivello F. Grassiot B. Alperovitch A. Tzourio C. Mazoyer B. (2005). Age- and sex-related effects on the neuroanatomy of healthy elderly. *NeuroImage* 26 900–911. 10.1016/j.neuroimage.2005.02.042 15955500

[B63] Lezak M. D. (1995). *Neuropsychological Assessment.* New York, NY: Oxford University Press.

[B64] Li K. Z. H. Bherer L. Mirelman A. Maidan I. Hausdorff J. M. (2018). Cognitive involvement in balance, gait and dual-tasking in aging: a focused review from a neuroscience of aging perspective. *Front. Neurol.* 9:913. 10.3389/fneur.2018.00913 30425679 PMC6219267

[B65] Lussier M. Renaud M. Chiva-Razavi S. Bherer L. Dumoulin C. (2013). Are stress and mixed urinary incontinence associated with impaired executive control in community-dwelling older women? *J. Clin. Exp. Neuropsychol.* 35 445–454. 10.1080/13803395.2013.789483 23656544

[B66] Lussier M. Saillant K. Vrinceanu T. Hudon C. Bherer L. (2020). Normative data for a tablet-based dual-task assessment in healthy older adults. *Arch. Clin. Neuropsychol.* 36 1316–1325. 10.1093/arclin/acaa121 33372951 PMC8517623

[B67] Mailey E. L. White S. M. Wojcicki T. R. Szabo A. N. Kramer A. F. McAuley E. (2010). Construct validation of a non-exercise measure of cardiorespiratory fitness in older adults. *BMC Public Health* 10:59. 10.1186/1471-2458-10-59 20144197 PMC2831835

[B68] Maldonado T. Orr J. M. Goen J. R. M. Bernard J. A. (2020). Age differences in the subcomponents of executive functioning. *J. Gerontol. B Psychol. Sci. Soc. Sci.* 75 e31–e55. 10.1093/geronb/gbaa005 31943092

[B69] Marks B. L. Madden D. J. Bucur B. Provenzale J. M. White L. E. Cabeza R. (2007). Role of aerobic fitness and aging on cerebral white matter integrity. *Ann. N. Y. Acad. Sci.* 1097 171–174. 10.1196/annals.1379.022 17413020

[B70] McAuley E. Szabo A. N. Mailey E. L. Erickson K. I. Voss M. White S. M. (2011). Non-exercise estimated cardiorespiratory fitness: associations with brain structure, cognition, and memory complaints in older adults. *Ment. Health Phys. Act* 4 5–11. 10.1016/j.mhpa.2011.01.001 21808657 PMC3146052

[B71] Mekari S. Dupuy O. Martins R. Evans K. Kimmerly D. S. Fraser S. (2019). The effects of cardiorespiratory fitness on executive function and prefrontal oxygenation in older adults. *Geroscience* 41 681–690. 10.1007/s11357-019-00128-5 31728899 PMC6885073

[B72] Milham M. P. Erickson K. I. Banich M. T. Kramer A. F. Webb A. Wszalek T. (2002). Attentional control in the aging brain: insights from an fMRI study of the stroop task. *Brain Cogn.* 49 277–296. 10.1006/brcg.2001.1501 12139955

[B73] Miller E. K. Cohen J. D. (2001). An integrative theory of prefrontal cortex function. *Annu. Rev. Neurosci.* 24 167–202. 10.1146/annurev.neuro.24.1.167 11283309

[B74] Mirelman A. Herman T. Brozgol M. Dorfman M. Sprecher E. Schweiger A. (2012). Executive function and falls in older adults: new findings from a five-year prospective study link fall risk to cognition. *PLoS One* 7:e40297. 10.1371/journal.pone.0040297 22768271 PMC3386974

[B75] Miyake A. Friedman N. P. Emerson M. J. Witzki A. H. Howerter A. Wager T. D. (2000). The unity and diversity of executive functions and their contributions to complex “Frontal Lobe” tasks: a latent variable analysis. *Cogn. Psychol.* 41 49–100. 10.1006/cogp.1999.0734 10945922

[B76] Nashiro K. Qin S. O’Connell M. A. Basak C. (2018). Age-related differences in BOLD modulation to cognitive control costs in a multitasking paradigm: global switch, local switch, and compatibility-switch costs. *Neuroimage* 172 146–161. 10.1016/j.neuroimage.2018.01.030 29414492

[B77] Nielen J. T. H. de Vries F. van der Velde J. Savelberg H. Schaper N. C. Dagnelie P. C. (2019). The association between β-Blocker use and cardiorespiratory fitness: the maastricht study. *J. Cardiovasc. Pharmacol. Ther.* 24 37–45. 10.1177/1074248418778551 29793358 PMC6297897

[B78] Petzinger G. M. Holschneider D. P. Fisher B. E. McEwen S. Kintz N. Halliday M. (2015). The effects of exercise on dopamine neurotransmission in Parkinson’s disease: targeting neuroplasticity to modulate basal ganglia circuitry. *Brain Plast.* 1 29–39. 10.3233/bpl-150021 26512345 PMC4621077

[B79] Prakash R. S. Voss M. W. Erickson K. I. Lewis J. M. Chaddock L. Malkowski E. (2011). Cardiorespiratory fitness and attentional control in the aging brain. *Front. Hum. Neurosci.* 4:229. 10.3389/fnhum.2010.00229 21267428 PMC3024830

[B80] Rogers R. L. Meyer J. S. Mortel K. F. (1990). After reaching retirement age physical activity sustains cerebral perfusion and cognition. *J. Am. Geriatr. Soc.* 38 123–128. 2299115 10.1111/j.1532-5415.1990.tb03472.x

[B81] Ross R. Blair S. N. Arena R. Church T. S. Despres J. P. Franklin B. A. (2016). Importance of assessing cardiorespiratory fitness in clinical practice: a case for fitness as a clinical vital sign: a scientific statement from the American Heart Association. *Circulation* 134 e653–e699. 10.1161/CIR.0000000000000461 27881567

[B82] Schaap L. A. Pluijm S. M. Deeg D. J. Visser M. (2006). Inflammatory markers and loss of muscle mass (sarcopenia) and strength. *Am. J. Med.* 119:526.e9–17. 10.1016/j.amjmed.2005.10.049 16750969

[B83] Shallice T. (1988). *From Neuropsychology To Mental Structure.* Cambridge: Cambridge University Press.

[B84] Shiffrin R. M. Schneider W. (1977). Controlled and automatic human information processing: II. Perceptual learning, automatic attending and a general theory. *Psychol. Rev.* 84:127.

[B85] Smith A. E. Evans H. Parfitt G. Eston R. Ferrar K. (2016). Submaximal exercise-based equations to predict maximal oxygen uptake in older adults: a systematic review. *Arch. Phys. Med. Rehabil.* 97 1003–1012. 10.1016/j.apmr.2015.09.023 26525524

[B86] Spirduso W. W. (1980). Physical fitness, aging, and psychomotor speed: a review. *J. Gerontol.* 35 850–865. 7002994 10.1093/geronj/35.6.850

[B87] Stuckenschneider T. Askew C. D. Rüdiger S. Polidori M. C. Abeln V. Vogt T. (2018). Cardiorespiratory fitness and cognitive function are positively related among participants with mild and subjective cognitive impairment. *J. Alzheimers Dis.* 62 1865–1875. 10.3233/JAD-170996 29614659

[B88] Sylvia L. G. Bernstein E. E. Hubbard J. L. Keating L. Anderson E. J. (2014). Practical guide to measuring physical activity. *J. Acad. Nutr. Diet* 114 199–208. 10.1016/j.jand.2013.09.018 24290836 PMC3915355

[B89] Szuhany K. L. Bugatti M. Otto M. W. (2015). A meta-analytic review of the effects of exercise on brain-derived neurotrophic factor. *J. Psychiatr. Res.* 60 56–64. 10.1016/j.jpsychires.2014.10.003 25455510 PMC4314337

[B90] Taconnat L. Lemaire P. (2014). Fonctions exécutives, vieillissement cognitif et variations stratégiques. *Psychol. Fr.* 59 89–100. 10.1016/j.psfr.2013.03.007

[B91] Tang A. Eng J. J. Tsang T. S. Krassioukov A. V. (2013). Cognition and motor impairment correlates with exercise test performance after stroke. *Med. Sci. Sports Exerc.* 45 622–627. 10.1249/MSS.0b013e31827a0169 23135375 PMC4492717

[B92] Unterrainer J. M. Rahm B. Kaller C. P. Leonhart R. Quiske K. Hoppe-Seyler K. (2004). Planning abilities and the Tower of London: is this task measuring a discrete cognitive function? *J. Clin. Exp. Neuropsychol.* 26 846–856. 10.1080/13803390490509574 15370380

[B93] van Boxtel M. P. Paas F. G. Houx P. J. Adam J. J. Teeken J. C. Jolles J. (1997). Aerobic capacity and cognitive performance in a cross-sectional aging study. *Med. Sci. Sports Exerc.* 29 1357–1365. 9346168 10.1097/00005768-199710000-00013

[B94] Vaughan L. Giovanello K. (2010). Executive function in daily life: age-related influences of executive processes on instrumental activities of daily living. *Psychol. Aging* 25 343–355. 10.1037/a0017729 20545419

[B95] Veldsman M. Tai X. Y. Nichols T. Smith S. Peixoto J. Manohar S. (2020). Cerebrovascular risk factors impact frontoparietal network integrity and executive function in healthy ageing. *Nat. Commun.* 11:4340. 10.1038/s41467-020-18201-5 32895386 PMC7477206

[B96] Verhaeghen P. Cerella J. (2002). Aging, executive control, and attention: a review of meta-analyses. *Neurosci. Biobehav. Rev.* 26 849–857. 12470697 10.1016/s0149-7634(02)00071-4

[B97] Verstynen T. D. Lynch B. Miller D. L. Voss M. W. Prakash R. S. Chaddock L. (2012). Caudate nucleus volume mediates the link between cardiorespiratory fitness and cognitive flexibility in older adults. *J. Aging Res.* 2012:939285. 10.1155/2012/939285 22900181 PMC3415086

[B98] Voss M. W. Soto C. Yoo S. Sodoma M. Vivar C. van Praag H. (2019). Exercise and hippocampal memory systems. *Trends Cogn. Sci.* 23 318–333. 10.1016/j.tics.2019.01.006 30777641 PMC6422697

[B99] Wang Y. Chen S. Lavie C. J. Zhang J. Sui X. (2019). An overview of non-exercise estimated cardiorespiratory fitness: estimation equations, cross-validation and application. *J. Sci. Sport Exerc.* 1 38–53. 10.1007/s42978-019-0003-x

[B100] Warren B. J. Dotson R. G. Nieman D. C. Butterworth D. E. (1993). Validation of a 1-mile walk test in elderly women. *J. Aging Phys. Act.* 1:13. 10.1123/japa.1.1.13

[B101] Wasylyshyn C. Verhaeghen P. Sliwinski M. J. (2011). Aging and task switching: a meta-analysis. *Psychol. Aging* 26 15–20. 10.1037/a0020912 21261411 PMC4374429

[B102] Weinstein A. M. Voss M. W. Prakash R. S. Chaddock L. Szabo A. White S. M. (2012). The association between aerobic fitness and executive function is mediated by prefrontal cortex volume. *Brain Behav. Immun.* 26 811–819. 10.1016/j.bbi.2011.11.008 22172477 PMC3321393

[B103] Wendell C. R. Gunstad J. Waldstein S. R. Wright J. G. Ferrucci L. Zonderman A. B. (2014). Cardiorespiratory fitness and accelerated cognitive decline with aging. *J. Gerontol. A Biol. Sci. Med. Sci.* 69 455–462. 10.1093/gerona/glt144 24192540 PMC3968827

